# Tumor Necrosis Factor-stimulated Gene-6 (TSG-6) Is Constitutively Expressed in Adult Central Nervous System (CNS) and Associated with Astrocyte-mediated Glial Scar Formation following Spinal Cord Injury*

**DOI:** 10.1074/jbc.M115.710673

**Published:** 2016-07-19

**Authors:** Vivien J. Coulson-Thomas, Mark E. Lauer, Sara Soleman, Chao Zhao, Vincent C. Hascall, Anthony J. Day, James W. Fawcett

**Affiliations:** From the ‡ John Van Geest Cambridge Centre for Brain Repair, The E. D. Adrian Building, Forvie Site, Robinson Way, University of Cambridge, Cambridge CB2 0PY, United Kingdom,; § Department of Biomedical Engineering, Cleveland Clinic Lerner Research Institute, Cleveland, Ohio 44195,; ¶ Wellcome Trust-Medical Research Council Cambridge Stem Cell Institute and Department of Clinical Neurosciences, Clifford Allbutt Building, University of Cambridge, Cambridge CB2 0AH, United Kingdom, and; ‖ Wellcome Trust Centre for Cell-Matrix Research, Faculty of Life Sciences, University of Manchester, Manchester M13 9PT, United Kingdom

**Keywords:** astrocyte, glycosaminoglycan, hyaluronan, inflammation, proteoglycan, glial scar

## Abstract

Tumor necrosis factor (TNF)-stimulated gene-6 (TSG-6) binds to hyaluronan and can reorganize/stabilize its structure, also enhancing the binding of this glycosaminoglycan to its cell surface receptor, CD44. TSG-6 is rapidly up-regulated in response to inflammatory cytokines protecting tissues from the damaging effects of inflammation. Despite TSG-6 treatment having been shown to improve outcomes in an experimental model of traumatic brain injury, TSG-6 expression has not been extensively studied in the central nervous system (CNS). We hereby analyzed the expression profile of TSG-6 in the developing CNS and following injury. We show that TSG-6 is expressed in the rat CNS by GFAP^+^ and CD44^+^ astrocytes, solely in the mature brain and spinal cord, and is not present during the development of the CNS. *TSG-6*^−/−^ mice present a reduced number of GFAP^+^ astrocytes when compared with the littermate *TSG-6*^+/−^ mice. TSG-6 expression is drastically up-regulated after injury, and the TSG-6 protein is present within the glial scar, potentially coordinating and stabilizing the formation of this hyaluronan-rich matrix. This study shows that TSG-6 is expressed in the CNS, suggesting a role for TSG-6 in astrocyte activation and tissue repair. We hypothesize that within this context TSG-6 could participate in the formation of the glial scar and confer anti-inflammatory properties. Further studies are required to elucidate the therapeutic potential of targeting TSG-6 after CNS injury to promote its protective effects while reducing the inhibitory properties of the glial scar in axon regeneration.

## Introduction

Two types of glial cells, microglia and astrocytes (1), are particularly involved in the regulation of inflammatory events in the central nervous system (CNS). Astrocytes have vital roles in maintaining CNS homeostasis; regulating ion concentrations and providing metabolic support for neighboring neurons; stabilizing synapses; supporting the neurovascular system, including maintenance of the blood-brain barrier; and producing the extracellular matrix (ECM).^4^ Astrocytes are activated into “reactive astrocytes” following insults to the CNS. Immediately after injury (acute phase), astrocytes in and surrounding the injury site become proliferative, undergo morphological changes, and up-regulate the production of extracellular proteins at which point they are the main components of the glial scar (2–7). The role of the glial scar on nerve regeneration is controversial. A plethora of studies have established that the glial scar is not permissive to axonal growth and therefore inhibits axon regeneration (5–7). However, recent studies have demonstrated that preventing the formation of the astrocyte scar after injury does not result in spontaneous nerve regrowth (8). Therefore, current studies are breaking the prevailing dogma that the glial scar inhibits regeneration and indicating that certain components in the glial scar aid nerve regrowth. Moreover, despite the detrimental effect the glial scar has on neuroregeneration, it also plays a fundamental role in rapidly repairing the blood-brain barrier and controlling inflammatory cell infiltration, thereby confining the damaging effects of the injury (9–11).

Tumor necrosis factor (TNF)-stimulated gene-6 (TSG-6) is an ∼35-kDa protein that is secreted by a wide range of cell types in response to inflammatory mediators; it was originally identified as a gene product induced in fibroblasts by TNF (12). TSG-6 contains a link module domain that mediates interaction with the polysaccharide hyaluronan (HA) (13, 14), which is a high molecular weight glycosaminoglycan composed entirely of repeating disaccharides of glucuronic acid and *N*-acetylglucosamine. HA is a ubiquitous component of the ECM, including in the CNS, where it is up-regulated after injury in the scar tissue (15). The gene expression of TSG-6 is tightly regulated, and it is generally not constitutively expressed in adult mammalian tissues; however, there are exceptions to this, such as in human epidermis (16), the islets of the pancreas in mouse and man (17, 18), and the human amniotic membrane (19). For example, in the epidermis, TSG-6 staining is associated with keratinocytes, melanocytes, and Langerhans cells, which are in close proximity to pericellular HA (16), and in the pancreas TSG-6 is expressed by α and β cells of the islet where it may participate in the formation/stabilization of HA networks within the extracellular matrix (17). Within the islet, intense TSG-6 staining is also associated with a subset of inflammatory cells (18). On this basis, there may be a tendency toward constitutive expression of TSG-6 in tissues of high metabolic activity, high oxidative stress, and/or inflammatory insults.

TSG-6 is stored in the secretory granules of neutrophils and mast cells and released in response to proinflammatory signals (20, 21). In many stromal cells (*e.g.* fibroblasts and smooth muscle cells), TSG-6 is rapidly up-regulated in response to inflammatory cytokines and certain growth factors (22). TSG-6 is also up-regulated by monocytes, macrophages, and myeloid dendritic cells upon stimulation with proinflammatory mediators (20). Consistent with this expression pattern, TSG-6 has been found to be associated with inflammation and inflammatory disease processes (23), being present in joint tissues from patients with arthritis (24, 25), blood vessels following injury (26), and serum during bacterial sepsis (27). There is a growing body of evidence to show that the primary function of TSG-6 is to protect tissues from the damaging and unwanted effects of inflammation and that many of the tissue-protective and anti-inflammatory activities of mesenchymal stromal cells are mediated by TSG-6 (28). For example, TSG-6 is a potent inhibitor of neutrophil migration (29) and can also suppress inflammatory signaling in tissue-resident immune cells (30). Some of the effects of TSG-6 on immune cell responses are CD44-dependent (30, 31) where this may be mediated through the direct cross-linking of HA by TSG-6, which is known to enhance HA/CD44 interactions on leukocytes (14, 31–34). The interaction of TSG-6 with HA, which has been extensively characterized at a biophysical and structural level, promotes TSG-6 oligomerization, allowing multiple polysaccharide chains to link together and the rigidification/condensation of HA-rich matrices (14, 32).

As well as its direct interaction with HA, TSG-6 also plays a well defined role in catalyzing the covalent transfer of heavy chains (HCs) from the serum-derived proteoglycan inter-α-inhibitor (IαI; a serine protease inhibitor) and the related pre-α-inhibitor (PαI) onto HA chains (35, 36). This HA modification occurs whenever HA, IαI/PαI, and TSG-6 meet, and recently divalent cations (Ca^2+^, Mg^2+^, and Mn^2+^) have been shown to have a key structural and functional role in the TSG-6-mediated transfer of HC from IαI onto HA (35, 37). For example, HA and TSG-6 levels are generally increased in tissues during inflammation (22, 27, 38), and IαI/PαI can leak into the tissues from the circulation due to increased vascular permeability. The formation of HC-HA complexes is believed to provide ECM stabilization through cross-linking mechanisms (35, 39) and to regulate the interaction/migration of leukocytes (40). In some contexts, HA-HC-containing matrices have been implicated as having anti-inflammatory and tissue-protective properties, *e.g.* in the amniotic membrane (41, 42) and when produced by mesenchymal stem cells (43). However, in other instances, their formation may contribute to pathology, *e.g.* in lung disease (44). HA is present in the extracellular compartment of most tissues, including the CNS, where it is up-regulated after injury in the scar tissue (15). The synthesis of HA is also often up-regulated in response to inflammation, tissue damage, or invasion by tumor cells or pathogens (45–48). Hyaluronidases, endoglycosidases expressed by mammalian cells, may break high molecular weight HA into low molecular weight HA; however, the transfer of HCs from IαI to HA, which cross-links HA chains, may protect HA from digestion.

TSG-6 also interacts with other ligands in addition to HA, including sulfated glycosaminoglycans (*e.g.* chondroitin sulfate (CS) and heparan sulfate) (49) and core proteins from CS proteoglycans (*e.g.* aggrecan and versican) (50, 51). Furthermore, TSG-6 binds to extracellular signaling molecules, such as bone morphogenetic proteins (52) and chemokines (29, 53). In the case of CXCL8, TSG-6 inhibits the interaction of this proinflammatory chemokine with cell surface heparan sulfate, providing a mechanism by which TSG-6 impairs neutrophil migration into tissues (29). This anti-inflammatory activity of TSG-6 has been suggested, for example, to contribute to the beneficial effects of recombinant TSG-6 administration after tissue damage and recovery of memory in a mouse model of traumatic brain injury (54).

Thus, TSG-6 has a wide range of biological activities that are potentially relevant to inflammation and tissue injury/regeneration in the brain and spinal cord. However, to date there has been little analysis of TSG-6 expression in neuronal tissues. The only study we are aware of (non-peer reviewed) analyzed TSG-6 expression in a murine model of transient focal cerebral ischemia; TSG-6 mRNA was significantly increased postreperfusion, and the elevated TSG-6 protein was associated with astrocytes surrounding the infarcted tissue (55).

We hereby show that TSG-6 is expressed constitutively in the adult CNS by GFAP^+^/CD44^+^ astrocytes. Our findings provide evidence that TSG-6 is not expressed during development of the CNS but does, however, play a role in astrocyte maturation and during pathogenesis. TSG-6 expression is greatly up-regulated following CNS injury and is present within the glial scar most likely bound to HA (or CS) chains. Thus, TSG-6 may coordinate assembly and stabilization of the HA-rich matrices that contribute to glial scar formation.

## Results

### 

#### 

##### TSG-6 Is Present in the Rat Brain

**FIGURE 1.**
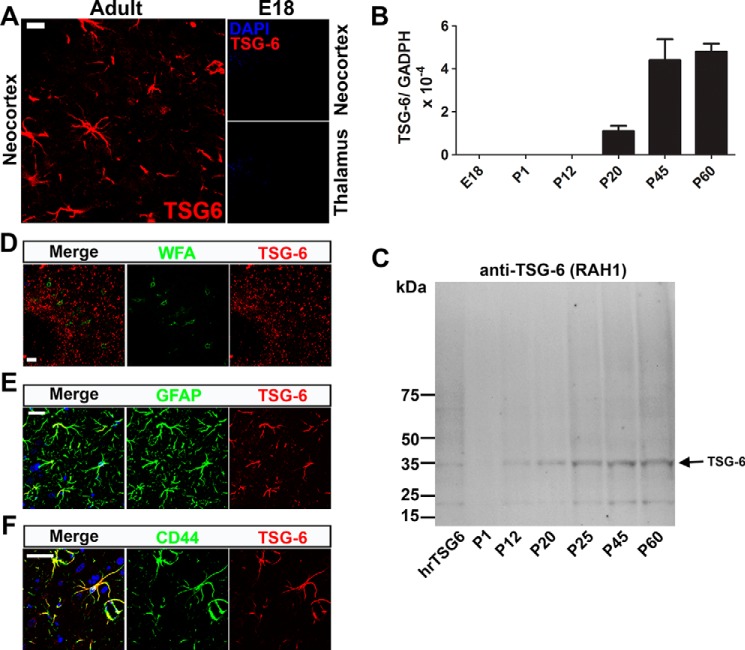
**TSG-6 is expressed in the mature CNS by astrocytes.** *A*, immunohistochemistry with anti-TSG-6 (RAH1) in the neocortex of rat brain tissue from adult (P60) and E18 embryos showed TSG-6 staining solely in the adult tissues in star-shaped cells with numerous projections. *B*, RNA was extracted from the brains of E18, P1, P12, P20, P45, and P60 rats, and TSG-6 expression was analyzed by real time PCR and normalized to the housekeeping gene *GADPH*; mean data are shown with *error bars* representing S.D. of differences between replicates (*n* = 9). *C*, proteins were extracted from P1, P40, and P60 rat brains and analyzed by Western blotting using an anti-TSG-6 antibody. Human recombinant TSG-6 (*hrTSG-6*) protein was used as control. *D*, co-staining with anti-TSG-6 and WFA showed that TSG-6 is not present in the perineuronal nets of adult rat brains (P60). *E*, co-staining with anti-TSG-6 and anti-GFAP identified astrocytes as the TSG-6-expressing cells in the CNS. *F*, co-staining with anti-TSG-6 and anti-CD44 antibodies revealed that CD44^+^ astrocytes express TSG-6. *Scale bars*, 20 μm.

TSG-6 expression in the CNS was analyzed in sections of adult (postnatal day 60 (P60)) rat brains by immunohistochemistry with anti-TSG-6 antibody. TSG-6^+^ cells had stellate morphology with various long cellular processes emerging from many sides of the cell body (Fig. 1*A*). A representative image of TSG-6^+^ cells in the neocortex is shown in Fig. 1; however, they were present throughout the brain tissue, adjacent to the meninges (pia mater) and associated with blood vessels. Because of the important role the ECM plays in CNS development and particularly during closure of critical periods of plasticity through the formation of the perineuronal nets, we looked for evidence of HA matrix stabilization by TSG-6 for the formation of HA network in the perineuronal nets during this period. TSG-6 staining was not found to co-localize with the perineuronal net marker *Wisteria floribunda* agglutinin (WFA) lectin in the brain (Fig. 1*D*, image of the neocortex). We investigated whether TSG-6 is also present during embryonic stages by immunostaining with anti-TSG-6 in sections of E18 and P1 rat brains; however, no positive staining was observed (Fig. 1*A* and results not shown, respectively).

The expression levels of TSG-6 were also analyzed by real time PCR of mRNA extracted from the brains of E18, P1, P12, P20, P45, and P60 rats. No TSG-6 mRNA was detected in the brains of rats at E18, P1, and P12. In contrast, TSG-6 expression was detected in the brains of rats at P20, P40, and P60 with an increase in TSG-6 expression in the latter time points (Fig. 1*B*). To confirm TSG-6 protein levels in the CNS, total protein was extracted from the brains of P1, P12, P20, P25, P45, and P60. TSG-6 was not detected in Western blots of the P1 brain, but a band of the expected molecular mass (∼35 kDa) was present in samples from P12 to P60 brains with a gradual increase according to age (Fig. 1*C*); such a TSG-6 species was also observed with the anti-TSG-6 antibody A38.1 (results not shown). In Fig. 1*C*, an ∼20-kDa species is apparent, perhaps representing the formation of a proteolytic cleavage product of TSG-6 in the tissue. Interestingly, TSG-6 protein was detected at P12 by Western blotting analysis, but mRNA was not detected by quantitative PCR. This could indicate that during the early postnatal period there are both low levels of TSG-6 expression and turnover, allowing the protein to accumulate in astrocytes. Thus, TSG-6 is expressed in the adult rat brain and not in the developing brain. This is consistent with previous findings that *TSG-6*^−/−^ mice present no CNS developmental abnormalities (56), and therefore we can infer that TSG-6 does not have a role in CNS development. Moreover, we saw no evidence for the association of TSG-6 with perineuronal nets, which are present from P30 to adulthood, so we can also infer that TSG-6 is unlikely to be involved in the control of CNS plasticity (WFA^+^ neurons; Fig. 1*D*).

##### TSG-6 Is Expressed in the CNS by Astrocytes

To determine which cell type(s) in the CNS expresses TSG-6, we immunostained sections with different markers. TSG-6 staining was not present in microglia (CD68^+^ cells; results not shown). However, TSG-6 staining co-localized with GFAP^+^ cells in the CNS, indicating that a subtype or subpopulation of astrocytes is the primary cell type synthesizing and secreting TSG-6 (Fig. 1*E*, image of the neocortex). Previous studies have demonstrated that a subpopulation of astrocytes expresses CD44 in the spinal cord, retina, and cortex (57–60). Moreover, CD44 has also been suggested as a marker for astrocyte precursor cells (61). Given that CD44 is a well established cell surface receptor for HA on this cell population, we determined whether TSG-6 was expressed preferentially by CD44^+^ astrocytes. Indeed, all TSG-6-expressing astrocytes are also CD44^+^ astrocytes (Fig. 1*F*, image of the neocortex).

##### TSG-6 Is Expressed by Mature Astrocytes

**FIGURE 2.**
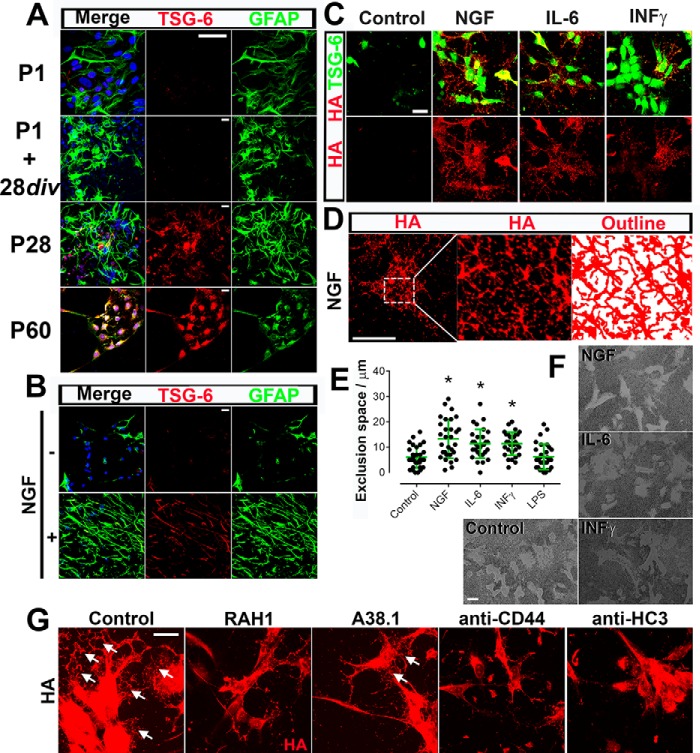
**TSG-6 is expressed by mature and activated astrocytes.** *A*, astrocytes were isolated from P1, P28, and P60 rat brains and analyzed by immunocytochemistry after 7 days in culture. The P1 astrocytes were also maintained for 28 div. The expression of GFAP and TSG-6 was analyzed in the astrocytes. Astrocytes isolated from P28 and P60 brains showed positive TSG-6 immunostaining, whereas those isolated from P1 rats, even when maintained for 28 div, were TSG-6-negative. *Scale bar*, 20 μm. *B*, the activation of astrocytes isolated from E18 brains with NGF for 48 h led to the expression of both GFAP and TSG-6. *Scale bar*, 20 μm. *C*, the activation of astrocytes with NGF, IL-6, or INFγ for 48 h led to the expression of TSG-6 and an organized netlike HA matrix. *Scale bar*, 20 μm. *D*, the outline of the organized HA matrix formed upon NGF activation was shown by tracing over the HA-positive labeling (using a MATLAB script), revealing an intricate fishnet design forming an organized three-dimensional HA network. *Scale bar*, 20 μm. *E*, the pericellular HA coats on live astrocytes activated or not with NGF, IL-6, or INFγ were quantified using the red blood cell exclusion assay. The widths (μm) of the pericellular matrix were measured as a depiction of the exclusion space for which mean values are represented graphically. *Error bars* represent S.D. *, *p* ≤ 0.05 compared with control. *F*, representative images demonstrate the exclusion width surrounding the activated astrocytes. *Scale bar*, 50 μm. *G*, astrocyte cultures isolated from E18 brains were cultured for 48 h in the presence of NGF with/without antibodies against TSG-6 (RAH1 and A38.1), HC3, and CD44. Formation of the HA netlike coats (*arrowheads*) was analyzed by immunofluorescence with biotinylated HABP (*red*), revealing HA and TSG-6 (RAH1; *green*). *Scale bar*, 20 μm.

We next investigated TSG-6 expression in primary astrocyte cultures prepared from P1, P28, and P60 rat brains and maintained *in vitro* for 48 h before fixation. Astrocytes obtained from P1 brains were also maintained in culture for 28 days *in vitro* (div) and then fixed. The astrocytes were stained with anti-GFAP and anti-TSG-6 antibodies. Adult astrocytes obtained from P28 and P60 brains presented strong TSG-6 expression. In contrast, the majority of the astrocytes isolated from embryonic brains presented no TSG-6 expression, and only ∼2% of the astrocytes presented weak staining for TSG-6 (Fig. 2*A*). Moreover, the astrocytes obtained from P1 brains and aged 28 div also presented weak TSG-6 staining in only ∼2% of the astrocytes (Fig. 2*A*). Because aging the astrocytes *in vitro* was not sufficient to lead to the expression of TSG-6, we attempted to induce TSG-6 expression by activating the astrocytes. Astrocytes were isolated from E18 brains, maintained *in vitro* for 5 days, and then cultivated in the presence or absence of NGF as an “activating factor” for 48 h (Fig. 2*B*). Indeed the increase in activated astrocytes was shown by the increase in GFAP^+^ cells present in astrocyte cultures grown in the presence of NGF, and these cells also stained positive for TSG-6 (Fig. 2*B*). Astrocytes maintained *in vitro* in the presence of another known activating factor, IL-6 or INFγ, for 48 h showed an essentially identical staining pattern with an equivalent increase in cells that were positive for both GFAP^+^ and TSG-6^+^ (Fig. 2*C* and results not shown). Astrocytes maintained in the presence of LPS showed an increase in GFAP^+^ cells but only a subtle increase in TSG-6^+^ astrocytes (results not shown).

We then investigated HA distribution in astrocytes that were or were not activated with NGF, IL-6, or INFγ. The exposure of astrocytes to NGF, IL-6, or INFγ led to the expression of TSG-6 and the formation of a dense and highly organized HA network associated with and surrounding the astrocytes and extending over the coverslip (Fig. 2*C*). NGF treatment yielded the greatest increase in HA distributed in this netlike manner. The morphology of the network of HA structures was further analyzed by automated tracing over the HA staining using a MATLAB script, which revealed the outline of this intricate network (Fig. 2*D*). HA cables clearly form a three-dimensional network resembling a fishnet design (Fig. 2*D*, *right panel*). The treatment of astrocytes with IL-6 or INFγ also led to the formation of an HA three-dimensional network, however not to the same extent as NGF (Fig. 2*C*). Some of the astrocytes that had not been exposed to NGF, IL-6, or INFγ (LPS-treated and control) presented subtle HA staining that was not organized in a netlike manner (results not shown and Fig. 2*C*, respectively). Therefore, the expression of TSG-6 correlated with the formation of a highly organized HA network formed by the activated astrocytes.

##### Pericellular Hyaluronan Coat on Live Activated Astrocytes

To further quantify the HA network surrounding astrocytes (*i.e.* treated with/without NGF, IL-6, or INFγ), the astrocyte pericellular HA coat was visualized using the red blood cell exclusion assay. The exclusion thickness surrounding the astrocytes, which represents HA-rich pericellular coats, was measured and averaged. Control astrocytes presented an exclusion thickness of ∼5 μm, whereas NGF, IL-6, and INFγ presented exclusion thicknesses of ∼12, 10, and 10 μm, respectively (Fig. 2, *E* and *F*).

##### Role of TSG-6 on HA Matrix Assembly

To elucidate the role of TSG-6 and other HA-binding proteins in the formation of the HA-rich matrix, astrocytes were activated with NGF in the presence of antibodies to neutralize TSG-6, HC3 (from PαI), and the hyaluronan receptor CD44. The anti-TSG-6 antibody A38.1 has been shown previously to inhibit HA/TSG-6 interactions (62) and impair HC-HA formation (63). We identified through proteomics that HC3 is present in the CNS and associated with HA^5^; therefore, an anti-HC3 antibody was used. Astrocytes were activated with NGF in the absence/presence of antibodies against TSG-6, HC3, and CD44. An intricate HA network can be observed around the control astrocytes stimulated with NGF alone (Fig. 2*G*, *arrows*). Astrocytes stimulated with NGF in the presence of anti-TSG-6 antibody RAH1, anti-CD44, and anti-HC3 antibodies presented HA on their surface but failed to form a highly organized HA matrix on the surface surrounding the cells (Fig. 2*G*). Astrocytes stimulated with NGF in the presence of anti-TSG-6 antibody A38.1 presented a drastic reduction of the highly organized HA matrix (Fig. 2*G*, *arrows*). Therefore, these data reveal that TSG-6, along with the cell surface receptor CD44 and HC3, are likely required for the formation of the three-dimensional HA network.

##### Role of TSG-6 in Astrocyte Maturation

**FIGURE 3.**
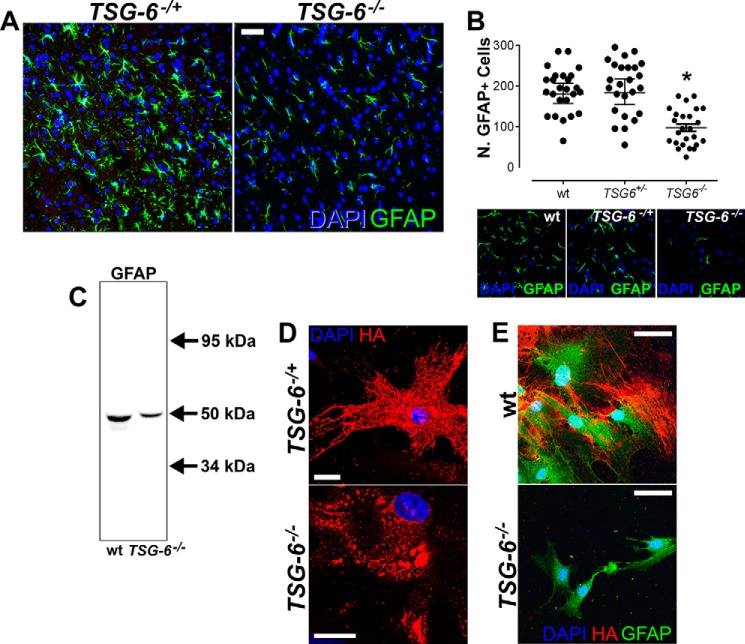
***TSG-6*^−/−^ mice have reduced numbers of GFAP^+^ astrocytes.** *A*, brain tissues (neocortex) from *TSG-6*^−/−^ mice and age-matched heterozygous littermates were analyzed by immunohistochemistry using anti-GFAP (*green*). DAPI was used to stain nuclei (*blue*). *Scale bar*, 20 μm. *B*, the scatter plot represents the number of GFAP^+^ cells in images captured from *TSG-6*^−/−^ and *TSG-6*^+/−^ mice. GFAP^+^ cells were counted in five images captured from both the neocortex and hippocampus from a total of seven different specimens. *Error bars* represent S.E. of the mean. *, *p* ≤ 0.05 for TSG6^−/−^ compared with either WT or TSG6^+/−^. *C*, cytoskeletal proteins were isolated from the brains of *TSG-6*^−/−^ and age-matched wild-type mice, and 12.5 μg of total protein was analyzed by Western blotting analysis to evaluate GFAP content. Anti-β-actin staining was used as a loading control. *D*, astrocytes were isolated from P1 brains of *TSG-6*^−/−^ and *TSG-6*^+/−^ mice and maintained in culture for 48 h in serum-free medium supplemented with NGF. The cells were stained with biotinylated HABP (*red*) to analyze the HA matrix produced by astrocytes. Nuclei were stained with DAPI (*blue*). *Scale bar*, 20 μm. *E*, astrocytes were isolated from 3-month-old *TSG-6*^−/−^ and age-matched wild-type mice and maintained in culture for 5 days with NGF added for the last 48 h. The cells were double stained with anti-GFAP and biotinylated HABP to analyze the HA matrix produced by astrocytes. Nuclei were stained with DAPI (*blue*). *Scale bar*, 20 μm.

To determine whether TSG-6 is necessary for astrocyte maturation, we evaluated the distribution of GFAP^+^ astrocytes in the neocortex and hippocampus of seven adult *TSG-6*^−/−^ mice compared with the littermate control heterozygous mice (*TSG-6*^+/−^). There was an overall decrease in the number of GFAP^+^ cells in both the neocortex and hippocampus of *TSG-6*^−/−^ mice (Fig. 3, *A* and *B*; images presented are of the neocortex). These data were confirmed by extracting total GFAP from three *TSG-6*^−/−^ and age-matched wild-type mice. Western blotting revealed a decrease in GFAP expression in the *TSG-6*^−/−^ mice when compared with age-matched wild-type controls (Fig. 3*C*). It is important to note that *TSG-6*^−/−^ mice have no CNS-related phenotype or macroscopic abnormalities, indicating that the lack of TSG-6 does not lead to any obvious developmental defect. However, the loss of TSG-6 does appear to hinder the maturation of astrocytes, revealing a role for TSG-6 in this process. To determine whether TSG-6 contributes to the organization of the dense HA matrix secreted by astrocytes activated with NGF, astrocyte cultures were isolated from both P1 and 3-month-old *TSG-6*^−/−^ and age-matched control mice. Astrocytes isolated from *TSG-6*^+/−^ mice produced an intricate three-dimensional HA network upon NGF stimulation; however, astrocytes isolated from *TSG-6*^−/−^ mice were unable to form such a matrix, and HA accumulated in clumps (Fig. 3*D*). Moreover, HA processes do not extend from the *TSG-6*^−/−^ astrocyte cell surface (Fig. 3*D*). Interestingly, when astrocytes were isolated from 3-month-old brains, only astrocytes isolated from the control wild-type mice produced a dense HA matrix upon NGF stimulation (Fig. 3*E*). It is important to note that isolating astrocytes from 3-month-old brains is challenging, and therefore astrocytes are exposed to harsh conditions from which potentially the *TSG-6*^−/−^ astrocytes do not recover from effectively hindering their HA expression.

##### Role of TSG-6 in the Reactive Glia and Scar Formation after Spinal Cord Injury in Rats

**FIGURE 4.**
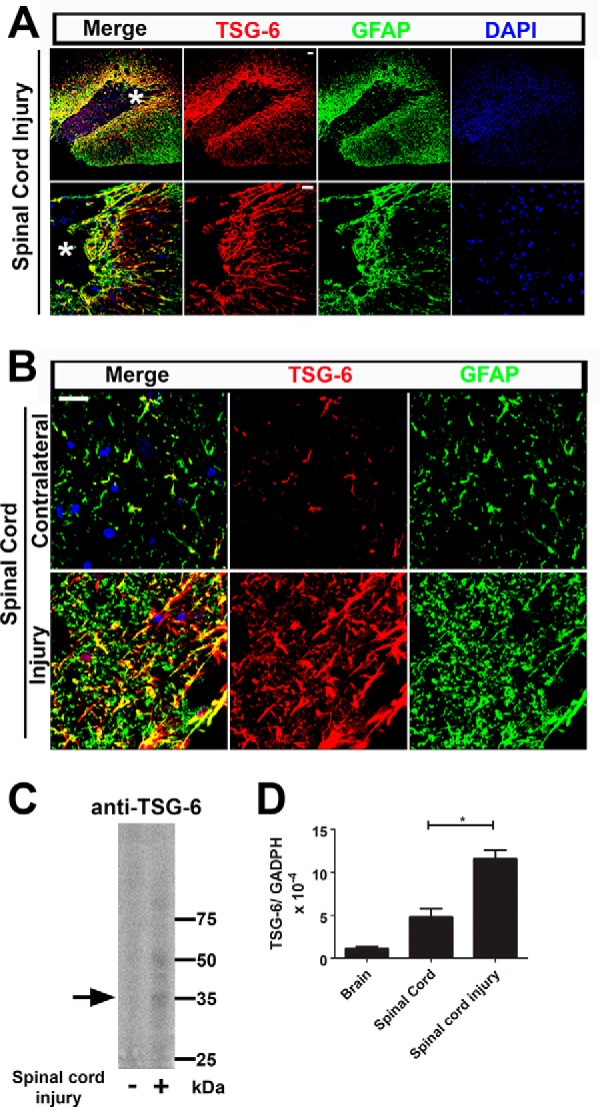
**TSG-6 is up-regulated after spinal cord injury.** *A*, TSG-6 and GFAP expression was analyzed after spinal cord injury. There is an increase in TSG-6 staining surrounding the injury site that is co-localized with GFAP immunoreactivity. TSG-6 was also present in the deposited ECM scar tissue. Images of the same area (indicated by an *asterisk* in the *first panel*) are presented with increased magnification in the *lower panels. Scale bar*, 100 μm. *B*, the localization of TSG-6 was compared at the injury site and in the contralateral side of the spinal cord. In the contralateral, uninjured spinal cord, TSG-6 staining is limited to GFAP^+^ cells. After spinal cord injury, TSG-6 expression is up-regulated and deposited in the matrix around the GFAP^+^ cells. Moreover, TSG-6 forms cable-like structures in the ECM perpendicular to the injury edge. *Scale bar*, 20 μm. *C*, proteins were extracted from injured and uninjured spinal cords and analyzed by Western blotting using an anti-TSG-6 antibody (RAH1), and anti-β-actin staining was used as a loading control. The *arrow* indicates TSG6. *D*, mRNA was extracted from injured and uninjured spinal cords, and TSG-6 expression was analyzed by real time PCR. *Error bars* represent S.D. *, *p* ≤ 0.05.

Astrocytes have a vital role in the CNS following injury. Astrocytes surrounding the injury site are activated into reactive glia that synthesize the ECM that later forms the glial scar. To determine whether TSG-6 is involved in the activation of astrocytes following spinal cord lesions and scar formation, spinal cord injuries were performed using the rat model. TSG-6 expression was up-regulated in the reactive glia in and surrounding the injury site and was also present in the scar tissue. The GFAP^+^ astrocytes, which were up-regulated within and surrounding the injury site, all presented TSG-6 staining (Fig. 4*A*). The ∼10-fold increase in TSG-6 expression surrounding the injury site was evident when compared with other regions of the spinal cord white matter that were uninjured (Fig. 4*B*). Controls with only secondary antibody were done and showed no staining (results not shown). Proteins and mRNA were extracted from the spinal cord injury site 10 days after the injury and from age-matched naïve spinal cords to evaluate TSG-6 deposition and expression. Indeed Western blotting analysis revealed an ∼10-fold increase in the amount of TSG-6 in the injured spinal cord when compared with the age-matched control tissue (Fig. 4*C*, *arrow*). The expression levels of TSG-6 10 days after spinal crush injury were also evaluated by real time PCR, which revealed an ∼2.5-fold increase when compared with control spinal cords from naïve littermates (Fig. 4*D*).

**FIGURE 5.**
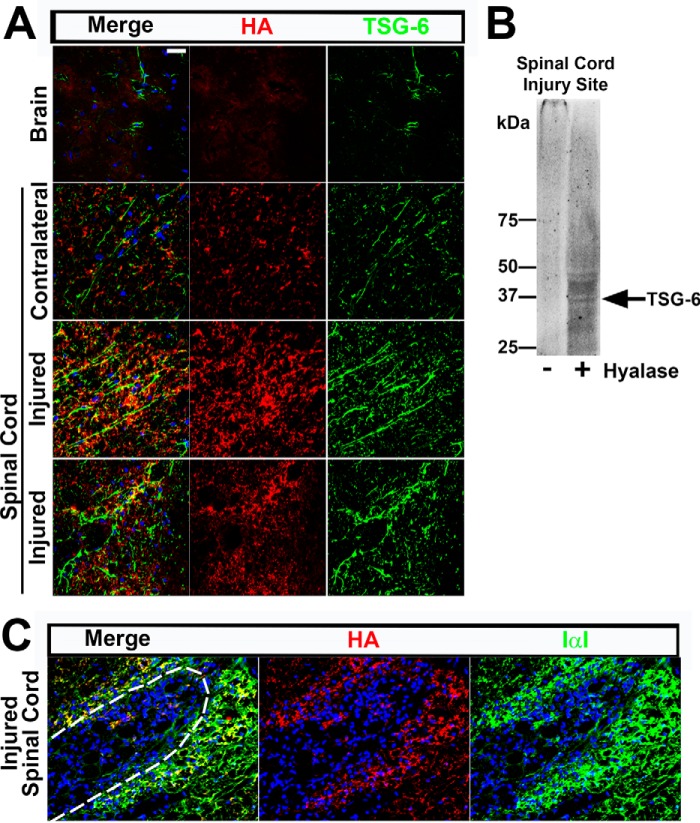
**TSG-6 is present in the HA-rich glial scar after injury.** *A*, TSG-6 and HA staining patterns were analyzed by histochemistry after spinal cord injury and compared with contralateral uninjured spinal cord and brain tissues (neocortex). HA is up-regulated after spinal cord injury and shows some co-localization with TSG-6 in the ECM. *B*, proteins were sequentially extracted from injured spinal cords with PBS, 1 m NaCl, and 7 m urea. The 7 m urea fraction was desalted and treated with/without hyaluronidase (*Hyalase*) and analyzed by Western blotting using an anti-TSG-6 antibody (RAH1), revealing TSG-6 species likely bound to HA in the glial scar. *C*, IαI/PαI staining patterns were analyzed by histochemistry after spinal cord injury and compared with contralateral uninjured spinal cord. IαI/PαI is up-regulated after spinal cord injury and shows some co-localization with HA in the ECM. *Dashed line* indicates the wound boundary. *Scale bar*, 20 μm.

Co-staining for TSG-6 and HA was done to determine whether TSG-6 was associated with HA in the glial scar. Indeed, TSG-6 was detected within the ECM co-localizing with HA as part of the scar tissue in the injured spinal cord (Fig. 5*A*, *lower two panels*). Moreover, long TSG-6^+^ cable-like processes were found extending perpendicularly from the wound edge into the scar tissue and extending from the tip of the injury site in the injured spinal cord (Fig. 5*A*, *third* and *fourth rows* of images, respectively). To confirm the presence of TSG-6/HA in the scar tissue, sequential protein extraction was done with buffers containing increasing salt concentrations to extract proteins present solely in the glial scar. The 7 m urea fraction, which would solubilize the HA-related matrix, was desalted and then analyzed with/without digestion with hyaluronidase. The untreated fraction contained a high molecular weight TSG-6 complex that was too large to enter the gel. After hyaluronidase digestion, several species immunoreactive with an anti-TSG-6 antibody were detected (Fig. 5*B*), including a band of ∼35 kDa. It is important to note that CS is susceptible to digestion by hyaluronidase from bovine testes and that CS also binds and interacts with TSG-6 (64). TSG-6 is therefore likely to be located in the glial scar bound to the HA- and/or CS-rich matrix. Interestingly, IαI/PαI was also detected in the glial scar co-localizing with HA (Fig. 5*C*). These data indicate that a specific cross-linked HA/TSG-6/HC matrix could also be present in the glial scar; however, further studies are required to investigate this. Thus, we have obtained evidence that TSG-6 has a role in astrocyte activation and may be an important constituent of the glial scar.

## Discussion

The results of this study demonstrate that TSG-6 is constitutively expressed by astrocytes in the adult brain and spinal cord. Moreover, we also found that TSG-6 expression is up-regulated after injury and present within the HA-rich glial scar.

In the CNS, there are many astrocyte morphologies, but traditionally there are two forms: the fibrous astrocytes, which are mainly located in the white matter, and the protoplasmic astrocytes, which are mainly located in the gray matter (65). GFAP, an intermediate filament protein, is a marker for terminally differentiated and reactive astrocytes and poorly labels unstimulated protoplasmic astrocytes. GFAP is expressed by fibrous astrocytes in late developmental stages and in reactive astrocytes following injury to the CNS (66, 67). Therefore, GFAP is commonly used as a marker for mature and/or “activated” astrocytes (68). Our findings show that TSG-6 is expressed in the rat CNS solely from around postnatal day 12 and is limited to GFAP^+^ astrocytes. This indicates that TSG-6 is constitutively expressed by mature astrocytes, and more importantly, that it may be involved in their maturation. Interestingly, it has been shown previously that a subset of GFAP^+^ astrocytes expresses the hyaluronan receptor CD44 (69, 70), and our findings show that TSG-6 is expressed by the same population of CD44^+^ astrocytes. Given that TSG-6 has been shown to stabilize HA-CD44 binding via HA cross-linking (32–34), in the CNS such an activity could alter the activated astrocyte state. Moreover, our data suggest that both CD44 and TSG-6 are required and collaborate in the formation of HA structures in the CNS. Furthermore, there is considerable evidence for the involvement of HA matrices in inflammatory processes (23, 43, 71–74). Interestingly, it has been shown that both T and B cells bind and migrate along cable-like HA structures in a CD44-dependent manner (75). Neutrophils and monocytes also migrate to inflammatory sites along HA chains via CD44/HA-dependent interactions (76). CD44 has also been shown to participate in the migration of leukocytes to inflammatory sites (77, 78). Moreover, TSG-6- and HA/TSG-6-rich matrices have also been shown to regulate inflammatory cell infiltration and activation (23, 43, 73, 79), such as via modulation of CD44/HA interactions (34).

TSG-6 is a tightly regulated protein, and its expression has been shown to be induced in fibroblasts, chondrocytes, monocytes, mesenchymal stem cells, and vascular smooth muscle cells upon stimulation by proinflammatory signals where there is little or no constitutive TSG-6 expression in most adult tissues (22, 80, 81). Our findings here show that the adult CNS contains astrocytes that constitutively express TSG-6 (mRNA and protein) and that these cells might contain a “storage pool,” which can be readily secreted upon injury. TSG-6 may therefore play a part in glial reactions in response to damage and inflammation. Immediately after spinal cord injury, it is important to limit inflammation and reconstitute the blood-brain barrier to limit tissue swelling and intracranial pressure, which could potentially cause further damage. A substantial number of studies have shown that TSG-6 has anti-inflammatory activity by directly regulating inflammatory cells and by regulating the assembly/organization of HA matrices, which have immunosuppressive properties (30–34, 39, 43, 80, 82–85). Moreover, HA has a well established role in the CNS and is a main constituent in the glial scar, which has recently been reviewed by our group (15). Therefore, based on the well characterized role TSG-6 has in inflammation, we hypothesize that the presence of TSG-6 in the glial scar could confer anti-inflammatory properties and therefore aid in reducing the inflammatory response after injury. There are various studies that demonstrate that astrocytes are involved in the control of inflammation around CNS injuries (10, 86). The presence of TSG-6 in a population of astrocytes in the healthy CNS may indicate that this immune suppressive activity is active in the normal CNS and poised to be rapidly available after damage.

Apart from a probable role in the control of inflammation, TSG-6 is involved in astrocyte maturation as demonstrated by the analysis of brains from *TSG-6*^−/−^ mice, which presented a decrease in the number of GFAP^+^ astrocytes. To investigate whether TSG-6 could also have a role in astrocyte activation (to reactive astrocytes), which takes place in response to CNS pathologies, such as stroke, trauma, or neurodegenerative disease, we investigated TSG-6 expression after activating astrocytes *in vitro*. When immature astrocytes (isolated from E18 brains) were treated with NGF, IL-6, or INFγ, within 48 h there was an increase in GFAP^+^ astrocytes and the induction of TSG-6 expression. Moreover, these activated astrocytes secreted a dense extracellular network of HA, particularly around NGF-treated astrocytes. Growing evidence suggests that neurotrophins not only signal neuronal cells but also exert important signaling cues in glial cells, including astrocytes (87–90). NGF has been shown to increase astrocyte cell proliferation in culture (91). Studies have also shown that astrocytes express p75^NTR^ (neurotrophin receptor) upon injury or after specific stimuli (92). Moreover, NGF has been shown to bind and signal through p75^NTR^ on astrocytes (93). These authors speculate that astrocyte-derived NGF may have an autocrine or paracrine effect on glial cells. A similar phenomenon has been shown for IL-6 where astrocytes present autocrine regulation of IL-6 and soluble IL-6 receptor (94). Interestingly, tropomyosin receptor kinases (Trks) (neurotrophin receptors) Trk A, Trk B, and Trk C are expressed in astrocytes in the mature CNS and are up-regulated after injury (95). Moreover, levels of neurotrophin receptors on astrocytes can be regulated by NGF (87). Our data corroborate the findings that NGF is an important signaling factor for astrocytes. We demonstrated that the activation of astrocytes with NGF leads to the formation of a dense HA matrix organized in an extensive netlike manner, which was not present around the untreated astrocytes. The activation of astrocytes with IL-6 or INFγ yielded similar results but to a lesser extent than NGF. LPS, which is commonly used to activate astrocytes, did not lead to the production of this HA-rich netlike matrix. Interestingly, astrocytes isolated from *TSG-6*-null mice treated with NGF failed to form the extensive HA network, indicating that TSG-6 has a key role in ECM assembly by reactive astrocytes and suggesting that the presence of CD44 is insufficient to support the formation of an HA network. Moreover, TSG-6 modulation of HA/CD44 interactions (32) may provide cues for regulating the activity of astrocytes by promoting the organization of the HA network. Also, astrocytes enmeshed within this HA matrix could behave differently in response to neurotrophic factors. Recently, IαI and pre-IαI have been reported in the CNS (96). Moreover, HC3 and HC4 were purified from CNS extracts using CS and HA affinity chromatography.^5^ In contrast, no HC1 or HC2 was detected. Here we demonstrate that the activation of astrocytes with NGF in the presence of anti-HC3 disrupts the assembly of the HA netlike matrix. Therefore, TSG-6 could potentially play a role in the transfer of HCs from IαI onto HA in the CNS. Thus, tailoring TSG-6 content after injury could enable control of the rigidification and condensation of the HA network and consequently the glial scar. Previous studies have also demonstrated that TSG-6 may participate in the incorporation of pentraxin 3 (PTX3) into the HA-HC matrices (39), and PTX3 has also been shown to be involved in glial scar formation (97). Therefore, TSG-6 could also participate in the formation of HA/HC/TSG-6/PTX3 matrices in the CNS in certain pathological conditions.

Following CNS pathogenesis, such as spinal cord injury, a dense meshwork of astrocytes, meningeal cells, and oligodendrocyte precursor cells homes into the injury site and become activated, constituting the glial reaction (5, 6). These activated astrocytes limit the inflammatory response and seal off the blood-brain barrier, providing neuroprotection (10, 98, 99) However, the activated astrocytes also secrete ECM components that are prominent in scar tissue. This scar tissue is composed primarily of CS proteoglycans, such as neurocan, versican, aggrecan, and brevican, which can bind to HA and form a dense matrix (100–106). Moreover, these HA-binding CS proteoglycans become more abundant during the weeks after injury (107) and can bind a number of inhibitory molecules and contribute to the failure of axonal growth through the scar tissue (6, 15, 108–112). TSG-6 also binds directly to CS chains and the G1 domains of aggrecan and versican (35, 38, 50, 51). TSG-6 has been suggested to antagonize the interaction of aggrecan with HA (50). Moreover, recent work shows that TSG-6 may also cross-link CS chains (64). TSG-6 can also mediate the transfer of HCs onto chondroitin (*i.e.* non-sulfated stretches of CS chains) (14, 38, 113). Given the well established role that TSG-6 has in regulating assembly of the HA matrices, including cross-linking HA chains, we investigated whether TSG-6 was involved in assembly of the glial scar after spinal cord injury and found that TSG-6 is up-regulated after spinal cord injury and is present both in reactive astrocytes and in the glial scar, co-localizing with extracellular deposition of the HA-specific matrix. Modulation of TSG-6 function is therefore a potential route to controlling the formation of glial scar tissue after CNS damage in cases of CNS inflammation in traumatic, infectious, and autoimmune conditions.

Taken together, these results demonstrate that TSG-6 has a significant role in astrocyte activation and in ECM deposition and assembly by reactive astrocytes. Moreover, TSG-6 expressed following injury could participate in suppressing neuroinflammation during the acute phase. Further studies are required to elucidate possible benefits of targeting TSG-6 expression and function after CNS pathology.

## Experimental Procedures

### 

#### 

##### Animals

For embryonic and postnatal brain collection at different time points, timed pregnant female Sprague-Dawley rats were purchased from Charles River Laboratories and maintained in the Brain Repair Centre in a temperature-controlled facility with an automatic 12-h light-dark cycle and food and water *ad libitum*. Transgenic *TSG-6*-null mice (Tnfip6^−/−^), hereafter referred to as *TSG-6*^−/−^ mice, were maintained in the Biological Resource Unit of the Cleveland Clinic Lerner Research Institute in a temperature-controlled facility with an automatic 12-h light-dark cycle and food and water *ad libitum* as approved by the Institutional Animal Care and Use Committee of the Cleveland Clinic. For experiments using *TSG-6*^−/−^ mice, both heterozygous littermate (*TSG-6*^+/−^) or wild-type (WT) mice were used as controls. For the spinal cord injury model using a single injection of d-lysophosphatidylcholine, Sprague-Dawley rats were maintained with standard husbandry at the Medical Research Council Research Centre in a temperature-controlled facility with an automatic 12-h light-dark cycle and food and water *ad libitum*. For the spinal cord crush injury model, Sprague-Dawley rats were maintained in the Brain Repair Centre in a temperature-controlled facility with an automatic 12-h light-dark cycle and food and water *ad libitum*. Experiments were done in accordance with the UK Home Office Regulations for the Care and Use of Laboratory Animals and the UK Animals (Scientific Procedures) Act 1986 and Cambridge University Guidelines.

##### Immunohistochemistry

Postnatal Sprague-Dawley rats (days 45 and 60; referred to as P45 and P60, respectively) were culled and perfused with 4% buffered paraformaldehyde, and thereafter their brains were removed. P1, P12, and P20 rats and 18-day (E18) Sprague-Dawley rat embryos were culled, and their brains were removed and immersion-fixed overnight at 4 °C. The brains were removed from three animals for each separate time point. After fixation, all brains were placed in 30% sucrose for 2 days, then mounted in freezing conditioning medium on dry ice, and stored at −80 °C. Sections 7 μm thick were cut with the use of a cryostat, mounted on silane slides, and stored at −20 °C. Upon use, the slides were incubated at room temperature for 2 h, and excess tissue conditioning medium was washed away with phosphate-buffered saline (PBS). Tissue sections were incubated for 1 min in 0.1 m glycine, washed with PBS, subsequently incubated for 1 h in blocking solution (containing 5% FBS and 0.01% saponin in PBS) at room temperature, and then incubated with primary antibodies overnight at 4 °C. Primary antibodies used were anti-GFAP (Abcam; at 1:500); rabbit anti-human polyclonal against TSG-6 (RAH1; at 1:250), which recognizes the TSG-6 C terminus (114); anti-TSG-6 monoclonal antibody (A38.1; at 1:250), which recognizes the link module domain of TSG-6 (62, 63); biotinylated WFA lectin (Sigma-Aldrich; at 1:100); anti-IαI antibody (Dako, A0301; at 1:100); and anti-CD44 (Abcam; at 1:100). Afterward, the tissue slices were washed three times in PBS and then incubated for 1 h at room temperature with appropriate fluorescent secondary donkey antibodies conjugated with Alexa Fluor® 488, Alexa Fluor 555, or Alexa Fluor 647 (Invitrogen). Thereafter, the tissue slices were washed with PBS, nuclei were stained with DAPI (Sigma), and coverslips were placed over the tissues with Fluoromount G (2:1 in PBS; Electron Microscopy Sciences) and sealed with nail polish. Tissue sections were examined using a Leica TCS SPE (Leica, Germany) inverted confocal microscope, and images were analyzed using Leica LAS LAF software.

##### TSG-6 RNA and Protein Extraction

Three separate brains from E18, P1, P12, P20, P25, P45, and P60 Sprague-Dawley rats were pooled for protein and RNA extraction. Proteins were extracted with radioimmune precipitation assay buffer containing protease inhibitors, and thereafter 15 μg of total protein was applied to NuPAGE 4–20% Bis-Tris gels (Invitrogen) and separated using a Novex® Mini-Cell (Invitrogen) system to evaluate TSG-6 expression. Proteins were transferred by electric current using an XCell II Blot Module (Invitrogen) to low fluorescence PVDF membranes (Bio-Rad), and TSG-6 was localized with anti-TSG-6 antibodies (RAH1 and A38.1) followed by donkey anti-rabbit or anti-rat antibody, respectively, conjugated with Alexa Fluor 488 and imaged using UVI (UVITEC, Alliance 4.7, UK). mRNA was extracted using the PureLink^TM^ RNA Mini kit (Ambion, Life Technologies) according to the manufacturer's instructions. cDNA was synthesized using SuperScript® III First-Strand (Invitrogen) according to the manufacturer's instructions. The primer combination used for quantitative PCR analysis of TSG-6 was as follows: forward, 5′ACGATGTCCACGGCTTTGTAGG3′; reverse, 3′GACGCATCACAAACTTCAAGG5′. The real time PCR was done using SYBR Green® and analyzed using a Bio-Rad CFX96 C1000 Thermal Cycler (Bio-Rad). Analysis of the data used the 2^−Δ*Ct*^ method with normalization to the housekeeping gene *GADPH* using the 7500 Real-Time PCR System software.

##### Astrocyte Cultures

Brains from P1, P28, or P60 Sprague-Dawley rats were collected and placed on wet ice until processing. Using a dissecting microscope, in Hanks' balanced salt solution initially, the meningeal membranes were removed using forceps and microscissors. Thereafter, the cerebellum and any remaining spinal cord were removed, and the remaining brain tissue was chopped into ∼2-mm pieces with the use of a blade. The P1 brains were digested with 0.1% trypsin for 30 min at 37 °C, whereas adult brains were digested in 0.1% trypsin with hyaluronidase from bovine testes (H3884, Sigma) for 45 min at 37 °C. The enzyme solutions were removed, Neurobasal medium (Life Technologies) was added, and the tissue was gently triturated with the use of a pipette. The minced tissue was centrifuged at 140 × *g* for 5 min, and the pellet was suspended in Neurobasal medium/DMEM (1:1) supplemented with 10% FBS. The excess myelin was removed from the adult brains with the aid of myelin removal beads (Miltenyi Biotec, UK). The cells were seeded on poly-d-lysine-coated flasks. After 24 h, the microglia- and oligodendrocyte precursor cell-rich supernatants were removed from the cultures by shaking for 4 h at 70 rpm at 37 °C and replaced with fresh medium. For immunocytochemistry, the astrocytes were harvested with trypsin/EDTA, seeded on glass coverslips previously treated with poly-d-lysine, and fixed after 24 h unless otherwise specified.

To induce astrocyte reactivity, astrocytes isolated from the brains of E18 rats were stimulated with NGF, IL-6, INFγ, or LPS, and both astrocyte activation and HA-specific matrix production were analyzed. For such, astrocytes were isolated and maintained in culture as described above for 5 days. On day 5, the medium was supplemented with/without NGF (50 ng/ml), IL-6 (50 ng/ml), INFγ (50 ng/ml), or LPS (100 ng/ml), and cells were cultured for 48 h. The cells were then fixed and stained with anti-GFAP, anti-TSG-6, and biotinylated HABP (Calbio chem; at 1:250) to visualize the HA matrix. Z-stacks of the three-dimensional network were captured; however, a single central panel has been displayed in the figures because the intricate detail in the HA network is not clear in the overlay projections of all images due to the sheer density of the network. The outline of the HA network was traced using a MATLAB script. For the neutralization assay, anti-TSG-6 (A38.1 and RAH1), anti-CD44 (ab41478, Abcam), and anti-HC3 (ITI H3, C-16, Santa Cruz Biotechnology) were used.

##### Red Blood Cell Exclusion Assay

Astrocytes were isolated from E18 rat brains, and after 15 div, they were activated with either IL-6, INFγ, or NGF for 48 h; control astrocytes received solely PBS. Thereafter, fresh rat red blood cell suspensions were prepared by three sequential PBS washes followed by centrifugation. They were then immediately placed over the astrocytes and left to settle for between 15 and 45 min at 37 °C and imaged with a FLoid Cell Imaging Station (Life Technologies). The experiment was done in six replicates and repeated twice. 30 images were obtained for each experimental condition to allow accurate measurement of the size of the HA-rich pericellular coat surrounding the astrocytes. The exclusion width of the pericellular matrix (*i.e.* the thickness measured perpendicular to the astrocyte cell membrane to that of the closest erythrocyte) was measured from five astrocytes per image in a total of 30 images; this analysis was conducted blinded.

##### TSG-6^−/−^ Tissue Analysis

Brains were collected from 6–8-week-old *TSG-6*^−/−^ mice and littermate controls (heterozygous) on a BALB/c background (56). The brains were fixed and processed as described above, and the numbers of mature astrocytes were detected by GFAP staining. The numbers of GFAP^+^ astrocytes were manually counted in five images captured from seven separate specimens (total of 30 images) using a blinded system.

##### Astrocyte Cultures from TSG-6^−/−^ Mice

Brains were removed from P1 and 3-month-old *TSG-6*^−/−^ mice or age-matched *TSG-6*^+/−^ or wild-type mice, respectively. Astrocytes were isolated as described above and seeded on glass coverslips previously treated with poly-d-lysine. The P1 astrocytes were maintained *in vitro* for 48 h in serum-free medium supplemented with NGF (50 μg/ml). The astrocytes isolated from 3-month-old brains were maintained *in vitro* for 5 days. To verify the role of TSG-6 in assembly of the HA matrix, the astrocytes were stimulated with NGF (50 μg/ml) for the last 48 h. The presence of the organized HA matrix was analyzed by immunocytochemistry using biotinylated HABP as described above. The absence of TSG-6 in the *TSG-6*^−/−^ isolated astrocytes was confirmed with rabbit anti-mouse anti-TSG-6 (RAM1) (115).

##### Analysis of GFAP Expression in TSG-6^−/−^ Brain Tissue

To confirm and provide quantitative data on the reduction of GFAP^+^ astrocytes, cytoskeletal proteins were extracted from the brains of 4-month-old *TSG-6*^−/−^ mice and age-matched wild-type mice as described above. Thereafter, 40 μg of total protein was analyzed by Western blotting as described above. GFAP was revealed using anti-GFAP followed by anti-chicken Alexa Fluor 488 (Invitrogen), and the loading control was stained with mouse anti-β-actin (ab6276, Abcam) followed by donkey anti-mouse Alexa Fluor 555.

##### Spinal Cord Injury Models

For immunohistochemistry analysis, a single injection of d-lysophosphatidylcholine (lysolecithin; Sigma-Aldrich) was administered into the mouse spinal cord to produce a small lesion site. The lesion was created in the spinal cord white matter of 8–10-week-old female Sprague-Dawley rats (Harlan) by injecting 1 μl of 1% lysolecithin directly into both the dorsal and ventral funiculi via a laminectomy of the first lumbar vertebra, which causes focal demyelination at the site of injection. Lesion sites from six separate animals were analyzed. At 15 days after the operation, animals were sacrificed by intravenous injection of pentobarbital and perfused with 4% paraformaldehyde in PBS. Spinal cords were dissected out and immersed in 20% sucrose for 24–48 h, embedded in Tissue-Tek® O.C.T. Compound (Sakura® Finetek), and stored at −80 °C. The tissues were coronally sectioned (12-μm-thick sections) and mounted on poly-l-lysine-coated slides. The slides were stored at −80 °C until used for immunostaining.

The spinal cord crush injury model was used for protein and mRNA extraction. Male Sprague-Dawley rats were anesthetized using isoflurane, and an insertion was made in the dorsal area. The spinal vertebrae were removed to expose the cervical (C6) spinal cord, and a dorsal column crush was done using fine forceps to create a spinal cord crush injury. Thereafter, the appropriate adjoining cervical muscle layers were closed using one to two internal sutures. The naïve control rats did not undergo any surgery. Animals were culled 10 days postsurgery, and the injury sites or equivalent areas in the naïve control rats were collected immediately and stored at −80 °C until processing. mRNA and protein extracted from the injury sites of five animals were analyzed and compared with equivalent areas of five control rats.

##### Spinal Cord Injury RNA, Protein, and Extracellular Matrix Extraction

The injured area of the spinal cord (∼2 mm in length) and the equivalent area in the naïve littermate controls were collected immediately after the rats were sacrificed and stored at −80 °C until processing. Proteins were extracted, and 12.5 μg was analyzed by Western blotting for TSG-6 expression as described above; mouse anti-β-actin followed by donkey anti-mouse Alexa Fluor 555 was used as an additional loading control. Western blotting analyses were repeated five times with consistent results. mRNA was extracted, cDNA was synthesized, and quantitative PCR was done as mentioned above.

The extracellular matrix was also extracted from the glial scar after a spinal cord crush. To isolate the extracellular matrix, which constitutes the tightly bound HA matrix, sequential protein extractions were prepared. Tissues from three separate rats were minced first in PBS and centrifuged, and the supernatants were removed. The pellets were then suspended in PBS containing 1 m NaCl, triturated, and centrifuged, and the supernatants were removed. Finally, the pellets were suspended in PBS containing 7 m urea, triturated, and centrifuged. Each extraction step was repeated twice before proceeding to the next buffer. The urea fraction was then desalted using Millicon units with a 3000-Da cutoff (Millipore) against PBS at 4 °C and digested or not with hyaluronidase from bovine testes. The samples were then analyzed by Western blotting as described above.

##### Statistics

Unless stated otherwise, values are presented as the mean and S.D. The difference between two groups was compared by means of the Student's *t* test. A *p* value of ≤0.05 was considered to be statistically significant. Statistical analysis was performed using the GraphPad Prism version 5 software package (GraphPad Software, San Diego, CA).

## Author Contributions

V. J. C.-T. conceived the idea for the project, conducted most of the experiments, analyzed the results, and wrote most of the paper; M. E. L. conducted experiments and analyzed the results. S. S. and C. Z. conducted experiments and approved the final version. V. C. H. analyzed the results and wrote the paper. A. J. D. conceived the idea for the project, analyzed the results, and wrote the paper. J. W. F. conceived the idea for the project, analyzed the results, and wrote the paper.

## Footnotes

5 V. J. Coulson-Thomas and J. W. Fawcett, unpublished data.

## Footnotes

4 The abbreviations used are:

ECM: extracellular matrix
TSG-6: tumor necrosis factor stimulated gene-6
GFAP: glial fibrillary acidic protein
HA: hyaluronan
HC: heavy chain
IαI: inter-α-inhibitor
PαI: pre-α-inhibitor
CS: chondroitin sulfate
WFA: *W. floribunda* agglutinin
E: embryonic day
P: postnatal day
div: days *in vitro*
Trk: tropomyosin receptor kinase
PTX3: pentraxin 3
Bis-Tris: 2-[bis(2-hydroxyethyl)amino]-2-(hydroxymethyl)propane-1,3-diol
HABP: HA-binding protein.
